# Antiparasitic activity in Asteraceae with special attention to ethnobotanical use by the tribes of Odisha, India

**DOI:** 10.1051/parasite/2018008

**Published:** 2018-03-12

**Authors:** Sujogya Kumar Panda, Walter Luyten

**Affiliations:** 1 Department of Zoology, North Orissa University, Baripada- 757003 India; 2 Department of Biology, KU Leuven, 3000 Leuven Belgium

**Keywords:** Asteraceae, *Plasmodium*, *Trypanosoma*, *Leishmania*, Odisha (India), antiparasitic drugs

## Abstract

The purpose of this review is to survey the antiparasitic plants of the Asteraceae family and their applicability in the treatment of parasites. This review is divided into three major parts: (a) literature on traditional uses of Asteraceae plants for the treatment of parasites; (b) description of the major classes of chemical compounds from Asteraceae and their antiparasitic effects; and (c) antiparasitic activity with special reference to flavonoids and terpenoids. This review provides detailed information on the reported Asteraceae plant extracts found throughout the world and on isolated secondary metabolites that can inhibit protozoan parasites such as *Plasmodium*, *Trypanosoma*, *Leishmania*, and intestinal worms. Additionally, special attention is given to the Asteraceae plants of Odisha, used by the tribes of the area as antiparasitics. These plants are compared to the same plants used traditionally in other regions. Finally, we provide information on which plants identified in Odisha, India and related compounds show promise for the development of new drugs against parasitic diseases. For most of the plants discussed in this review, the active compounds still need to be isolated and tested further.

## Abstract (fr)

Le but de cette revue est d’étudier les plantes antiparasitaires de la famille des Asteraceae et leur applicabilité dans le traitement des parasites. Cette revue est divisée en trois parties principales: (a) littérature sur les utilisations traditionnelles des Asteraceae pour le traitement des parasites; (b) description des principales classes de composés chimiques des Asteraceae et leurs effets antiparasitaires; (c) activité antiparasitaire avec référence spéciale aux flavonoïdes et aux terpénoïdes. Cette revue fournit des informations détaillées sur les extraits d’Asteraceae rapportés à travers le monde et sur des métabolites secondaires isolés qui peuvent inhiber les parasites protozoaires, tels que *Plasmodium*, *Trypanosoma*, *Leishmania* et les vers intestinaux. En outre, une attention particulière est accordée aux Asteraceae d’Odisha (Inde), utilisées par les tribus locales comme antiparasitaires. Ces plantes sont comparées aux mêmes espèces utilisées traditionnellement dans d’autres régions. Enfin, nous fournissons des informations sur les plantes identifiées à Odisha et les composés qui seraient prometteurs en tant que médicaments candidats contre les maladies parasitaires. Pour la plupart des plantes discutées dans cette revue, les composés actifs doivent encore être isolés et testés plus avant.

## Introduction − Antiparasitic research

Parasite diseases are a major source of disease in both humans and animals and result in significant economic losses. Protozoan parasites threaten the lives of billions of people worldwide and are associated with significant morbidity and large economic impacts [88]. The lack of proper vaccines and the emergence of drug resistance make the search for new drugs for treatment and prophylaxis more urgent, including from alternative sources like plants. In 2005, Pink *et al.* published a review emphasizing that new antiparasitic drugs are urgently needed to treat and control diseases such as malaria, leishmaniasis, sleeping sickness and filariasis [124]. The discovery of quinine from *Cinchona succirubra* (Rubiaceae) and its subsequent development as an antimalarial drug represent a milestone in the history of antiparasitic drugs from nature. The 2015 Nobel Prize in Physiology or Medicine was awarded for the discovery of artemisinin and avermectin, which fundamentally changed the treatment of parasitic diseases around the globe. Both compounds are natural products, once again showing that nature can be a powerful source of medicines. A breakthrough for the development of antimalarial drugs was the identification of the sesquiterpene artemisinin from *Artemisia annua* (Asteraceae), which can even kill multidrug-resistant strains of *Plasmodium falciparum* [3,62]. Several semisynthetic derivatives of artemisinin (*e.g.*, the water-soluble artesunate) have been developed and are used in clinical practice today [62].

There are three major protozoan parasitic infections, caused by *Plasmodium*, *Leishmania* and *Trypanosoma* species. *Plasmodium* is the most significant of the protozoan parasites that infect humans. Found in tropical and sub-tropical regions of the world, malaria parasites threaten the lives of 3.3 billion people and cause 0.6–1.1 million deaths annually [70]. Six species of *Plasmodium* are responsible for causing malaria in humans [144], with *Plasmodium falciparum* and *Plasmodium vivax* being the most common and major causes. Leishmaniasis is caused by *Leishmania* sp., generating 1–1.5 million new cases annually [104]. The disease is endemic in 98 countries and is one of the neglected tropical diseases where the majority of the affected individuals are rural, underprivileged, and economically disadvantaged. African sleeping sickness (trypanosomiasis), is caused by two parasitic protozoans: *Trypanosoma brucei gambiense* (West Africa) and *Trypanosoma brucei rhodesiense* (East Africa) [15]. African trypanosomiasis threatens the lives of approximately 60 million people in sub-Saharan Africa and is fatal if untreated [70]. Another species of *Trypanosoma* (*T. cruzi*) is responsible for Chagas disease (American trypanosomiasis), and threatens the lives of millions primarily in Mexico, Latin America and the United States. The World Health Organization estimates that 8–10 million people are infected annually. There is also no vaccine for Chagas disease and no clinical trials of new drugs are under way; current treatment depends on only two chemotherapeutics − benznidazole and nifurtimox.

## Medicinal uses of Asteraceae with special reference to the tribes of Odisha (Orissa), India

The family Asteraceae (Compositae) is also known as the daisy family, sunflower family or thistle family. Asteraceae is derived from the term “aster” meaning “star” in Latin, and refers to the characteristic inflorescence with flower heads composed of florets (small flowers), and surrounded by bracts [12]. The family Asteraceae is one of the largest families comprising 1600–1700 genera and 24,000–30,000 species [30]. The family has 12 subfamilies and 43 tribes, and is distributed worldwide [16], but is most abundant in the temperate and warm-temperate regions. Most of the species are herbs and shrubs, while trees are fewer in number. Asteraceae have been commonly used in the treatment of various diseases since ancient times, as attested by classical literature. For this review, we collected literature from scientific journals, books, theses and reports *via* a library and electronic search (using databases viz. PubMed, Google Scholar and Scopus). Several researchers have systematically investigated Asteraceae for their therapeutic utility. More than 7000 compounds have already been isolated, and 5000 have been identified from this family, often associated with some bioactivity [3]. Members of the Asteraceae are claimed to have various properties: antipyretic, anti-inflammatory, detoxifying, antibacterial, wound-healing, antihemorrhagic, antalgic (also for headaches), anti-spasmodic, and anti-tussive, and have been considered beneficial for flatulence, dyspepsia, dysentery, lumbago, leucorrhoea, hemorrhoids, hypotension, and most importantly, some are hepatoprotective, antitumor and antiparasitic [68]. The majority of studies on Asteraceae throughout the world have focused on chemical analysis (nearly 7000 compounds already isolated). There are many papers on *in vitro* studies, especially on antimicrobial, antioxidant and anticarcinogenic properties, using selected cells and crude extracts or purified compounds. In the few published reviews on pure compounds, the structure-activity relations were studied as well as their mechanism of action. Despite the discovery of a large number of compounds in Asteraceae around the world, and the reported antiparasitic properties of members of the Asteraceae family, not many bioactivity studies on Asteraceae species have yet been carried out. In India, the family is represented by 900 species from 167 genera.

Due to their bioactive properties, plants from the Asteraceae family are commonly used in the traditional treatment of various diseases (Table 1). For instance, *Ageratum conyzoides* has been commonly used in India including in the state of Odisha, where the plant is traditionally used for diarrhoea, dysentery, intestinal colic [118] and malaria. This plant is well-known for the presence of phytochemicals such as alkaloids, coumarins, flavonoids, benzofurans, sterols and terpenoids, with the following identified compounds: friedelin, various sterols (including β-sitosterol and stigmasterol), various flavonoids, caryophyllene, coumarin, quercetin, as well as fumaric and caffeic acid [51]. *Bidens* *pilosa* is also found in Odisha, and is moreover widely used as folk medicine by indigenous tribes of the Amazon in the treatment of malaria [13]. About 201 compounds comprising 70 aliphatics, 60 flavonoids, 25 terpenoids, 19 phenylpropanoids, 13 aromatics, 8 porphyrins, and 6 other compounds, have been identified from this plant, as compiled previously [67]. However, the relation between *Bidens* *pilosa* phytochemicals and various bioactivities is not yet fully established, and should become a future research focus [7]. *Blumea* *lacera* is used for the treatment of all kinds of fever, including malaria, and contains phytocompounds such as fenchone, coniferyl alcohol derivatives, campesterol, flavonoids, lupeol, hentriacontane, hentriacontane, α-amyrin, β-sitosterol and triterpenes [7,80,105]. *Calendula officinalis* has found many medicinal applications and contains various terpenoids (sitosterols, stigmasterols, erythrodiol, brein, ursadiol and its derivatives; several triterpene glycosides like calendulaglycoside A; glucosides of oleanolic acid, etc.), various flavonoids (quercetin, isoquercetin, isorhamnetin-3-O-β-D-glycoside, narcissin, calendoflaside, calendoflavoside, calendoflavobioside, rutin, quercetin-3-O-glucoside and quercetin-3-O-rutinoside), coumarins, saponins and quinones [87].

**Table 1:** Traditional uses of plants of the Asteraceae family

Plant^1^	Traditional uses by the tribes of Odisha	Other parts of India/world
*Ageratum* *conyzoides* (L.) L.	Herb infusion is given for gastrointestinal ailments such as diarrhoea, dysentery and intestinal colic with flatulence [117,120]. Cold decoctions from the aerial parts are used to cure malarial fever (unpublished observations).	As worm medicine in Cameroon [157].

*Bidens* pilosa L.	Fresh juice from the aerial parts is used for intestinal worm infections, abdominal pain and stomach ache (unpublished observations).	Juice form the root and whole plant is used for the treatment of malaria (Africa, China) [142,157]. Whole plant is used by the Bukusu community of Kenya for tick prevention and control on livestock [159].

*Blumea* *lacera* (Burm.f.) DC.	The tribes use fresh leaf juice of this plant for the treatment of all kinds of fever, including malaria (unpublished observations).	Leaf juice is used to kill worms in children by the tribes of Madhya Pradesh, India [136].

*Calendula* *officinalis* L.	Cold decoction of leaf is used for amoebic and bloody dysentery (unpublished observations).	Flowers are used for the treatment of intestinal worms and amoebal infections in pets and pigs in British Columbia, Canada [64].

*Caesulia* *axillaris* Roxb.	Whole plant extract is given to cure malaria [113].	The whole plant is crushed and juice is extracted, which is given orally three times a day, along with curd to cure amoebic dysentery by the tribes of Madhya Pradesh, India [155].

*Centipeda* *minima* (L.) A. Braun & Asch.	Root decoction is used for the treatment of all kinds of fever [112]. Leaf decoction is commonly used for hookworm and roundworm (unpublished observations).	In China, decoction from whole plant is used for malaria treatment. The seed or dried aerial parts are used as a vermifuge and amoebicide (http://uses.plantnet-project.org/en/Centipeda_minima_(PROSEA).

*Eclipta* *alba* (L.) Hassk.	Treatment of malaria [112].	Leaf decoction is used by the Rakhain tribal healers of Chittagong Division, Bangladesh for the treatment of malaria [46].

*Eclipta* *prostrata* (L.) L. is a synonym of *Eclipta* *alba* (L.) Hassk.	Treatment of malaria: decoction of dried leaf with tea leaf tincture is administered orally twice a day for five days [118].	Infusion or juice of the plant mixed with honey is given for the treatment of malaria by the tribal communities of Pakistan [86].

*Elephantopus* *scaber* L.	Treatment of malaria: paste prepared from fresh root is taken orally once a day for three days [118]. Juice of leaf is used in the treatment of malaria [53].	Decoction from aerial parts is used to treat malaria by the tribes of Madagascar [86].

*Sphaeranthus* *indicus* L.	Helminths: whole plant paste with a pinch of salt is taken as an anthelmintic [107].	Root and seed powder is given orally to kill intestinal worms in children [39]. Whole plant paste with a pinch of common salt is taken as an anthelmintic [61].

*Tagetes* *erecta* L.	Cold decoctions of leaf and flower are used for all kinds of worm infections and dysentery (unpublished observations).	Plants used by native Amazonian groups from the Nanay River (Peru) for the treatment of malaria [61].

*Tridax* *procumbens* (L.) L.	Decoction prepared from leaves of *Tridax* *procumbens* and *Andrographis* *paniculata* (Burm. f.) Nees is used for the treatment of malaria fever (unpublished observations).	Used for the treatment of malaria by the tribes of Ghana [59], and Kwale community of the Kenyan Coast [90].

*Vernonia* *anthelmintica* (L.) Willd. This name is a synonym of *Baccharoides* *anthelmintica* (L.) Moench. and *Centratherum anthelminticum* (L.) Kuntze	Fruit powder is used in malaria fever, and for stomach ache during amoebic dysentery [81]. Seeds are used as an anthelmintic, especially in children (2-5 g with water on an empty stomach twice a day for three days) [111,112].	The seeds are used as an anthelmintic against parasitic worm (including tapeworm) infestations [4].

*Vernonia* *albicans* DC. This name is a synonym of *Cyanthillium* *albicans* (DC.) H. Rob.	Filariasis: powdered plant (10-20 g) is advised to be consumed with 125 mL milk (mixed with 5-7 cardamom fruits and 10 g sugar candy) once daily in the morning, on an empty stomach, for about three months [37]. Water-extract of the whole plant is used in the treatment of malaria [53].	–

*Vernonia* *cinerea* (L.) Less. This name is a synonym of *Cyanthillium* *cinereum* (L.) H. Rob.	Treatment of malaria; root paste is mixed with honey and administered orally twice a day for three days [118]. The plant is also used for elephantiasis [120].	Leaf and bark are used by the tribes of Equatorial Guinea as febrifuge and vermifuge [2], while the tribes of Tanzania use it for the treatment of malaria [84].

*Xanthium* *strumarium* L.	Coastal tribes of Odisha use crushed fresh fruit for the treatment of *filariasis* (unpublished observations).	Tribes of Bannu district, Pakistan, use it for the treatment of chronic malaria [154].

1 All taxonomic names were verified in the Global Composite Checklist database (http://compositae.landcareresearch.co.nz/Default.aspx)

Whole plant extracts of *Caesulia* *axillaris* are frequently used by the coastal tribes of Odisha to cure malaria [107,113], but no scientific studies have yet been published on this plant. *Centipeda* *minima* is widely distributed in Odisha, and is frequently used by the local tribes for the treatment of parasites [112], but no compounds responsible for its antiparasitic activities have yet been identified. *Eclipta* *prostrata* (synonym *E.* *alba*) is frequently used by the tribes for the treatment of malaria [113,130]. The plant is well studied for its phytochemistry, with documented presence of compounds such as eclipline, β-amyrin, luteolin-7-O-glucoside, apigenin, cinnaroside, stigmasterol, wedelolactone, columbin, triterpene glycosides and triterpenic acid [47]. Like *Eclipta* *prostrata, Elephantopus* *scaber* is also frequently used by the tribes for the treatment of malaria [118]. This plant is also well studied for its phytochemistry with documented presence of sesquiterpenelactones such as elescaberin, deoxyelephantopin, isodeoxyelephantopin, scabertopin, and isoscabertopin, and lipids like ethyl hexadecanoate, ethyl-9, 12-octadecadienoate, ethyl-(Z)-9-octadecenoate, ethyl octadecanoate, lupeol and stigmasterol [19]. Whole plant paste of *Sphaeranthus* *indicus* with a pinch of salt is taken as an anthelmintic by the tribes of Odisha [111]. The phytochemical studies of this plant suggest the presence of eudesmanolides, sesquiterpenoids, sesquiterpene lactones, sesquiterpene acids, flavone glycosides, flavonoid C-glycosides, isoflavone glycosides, sterols, sterol glycosides, alkaloids, peptide alkaloids, amino acids and sugars [125]. The essential oil from this plant has been well studied with the documented presence of bioactive compounds like sphaeranthine, sphaeranthol, spharerne, methyl chavicol, ocimene, geraniol, and methoxy frullanolides [71]. *Tagetes* *erecta* is an ornamental plant of Odisha and is often used by the tribes for the treatment of various conditions such as anaemia, irregular menstruation, abdominal pain, colic, cough and dysentery. Like *Sphaeranthus indicus*, this plant is also well known for its phytoconstituents such as β-sitosterol, β-daucosterol, 7-hydroxy sitosterol, lupeol, erythrodiol, erythrodiol-3-palmitate, quercetagetin, quercetagetin-7-methyl ether, quercetagetin-7-O-glucoside, gallic acid, syringic acid, quercetin, ocimene and tagetone [135]. *Tridax procumbens* has been extensively used in Ayurvedic medicine and is well-studied for its phytochemistry, with the presence of compounds like 8,3′-dihydroxy-3,7,4′-trimethoxy-6-O-β-D glucopyranoside flavonol, apigenin-7-O-β-D-glucoside, pentadecane, β-sitosterol, stigmasterol, β-daucesosterol and bis-(2-ethylhexyl)-phthalate [131]. Several species of *Vernonia* have been used in different traditional medicines all over the world. The tribes of Odisha most frequently use different species of *Vernonia: V.* *anthelmintica, V. albicans* and *V. cinerea.* Seeds of *Vernonia* *anthelmintica* are used as an anthelmintic, especially in children: 2-5 g with water on an empty stomach twice a day for three days [111,112]. Fruit powder is used in malaria fever, and stomach ache during amoebic dysentery [81]. Powdered *Vernonia* *albicans* plant (10-20 g) is advised to be consumed with 125 mL milk (mixed with 5-7 cardamom fruits and 10 g sugar candy) once in the morning, on an empty stomach for about three months for the treatment of filariasis [37]. The aqueous extract of the whole plant is also used in the treatment of malaria [53]. Root paste of *Vernonia* *cinerea* mixed with honey is administered orally twice a day for three days for malaria [108]. Reports are also available on the use of this plant for the treatment of elephantiasis [108]. Toyang and Verpoorte [152] published a review article on this genus *Vernonia* (109 species) concerning its ethnopharmacology and phytochemistry. *Xanthium strumarium* is a weed, widely distributed in Odisha, and commonly used as a medicinal plant. Most of its pharmacological effects can be explained by constituents like sesquiterpene lactones, glycosides, phenols, as well as polysterols present in all plant parts. The bioactive compounds reported for this plants are xanthinin, xanthumin, xanthatin (deacetylxanthinin), a toxic principle, namely a sulphated glycoside: xanthostrumarin, atractyloside, carboxyatractyloside, phytosterols, xanthanol, isoxanthanol, xanthinosin, 4-oxo-bedfordia acid, hydroquinone, xanthanolides, caffeoylquinic acids, α- and γ-tocopherol, thiazinedione and deacetyl xanthumin, β-sitosterol, γ-sitosterol, β-D-glucoside of β-sitosterol; isohexacosane, chlorobutanol, stearyl alcohol, stromasterol and oleic acid [52].

## Miscellaneous antiparasitic properties of Asteraceae and their phytochemistry

Over the past decades, a lot of research on antiparasitic drugs of plant origin has yielded undisputable metabolites of interest. Many plant-derived secondary metabolites of Asteraceae have exhibited target-specific activity against *Plasmodium*, *Leishmania* and *Trypanosoma* parasites (Table 2). Plants from the Asteraceae family are widely used as medicines due to the presence of a broad range of bioactive metabolites such as alkaloids (pyrrolizidine and pyridine), flavonoids, phenolic acids, coumarins, terpenoids (monoterpenes, sesquiterpenes, diterpenes, and triterpenes), quinoline and diterpenoid types, triterpenoid sesquiterpene lactones, pyrethrins, and saponins. Several sesquiterpenes have been reported as antiprotozoal since the discovery of artemisinin. The sesquiterpene lactone parthenin is effective against *Plasmodium falciparum* *in vitro*, with an EC_50_ value of 1.29 µg/mL [123]. Parthenin is capable of blocking parasite-specific targets responsible for glutathinonylspermidine and trypanothione synthesis from cysteine and glutathione precursors in both *Leishmania* and *Trypanosoma* [32]. The sesquiterpene lactones brevilin A from *Centipeda minima* and dehydrozaluzanin C from *Munnozia maronii* were discovered and reported as antiparasitic. Similarly, sesquiterpene lactones from *Neuroleaena lobata* are well established for the treatment of *Plasmodium* infections [28]. In this plant, structure-activity relationship analysis revealed that germanocrenolide sesquiterpenes, like neurolenin A (EC_50_ = 0.92 µM) and B (EC_50_ = 0.62 µM), were more potent than furanoheliangolides like lobatin A and B (EC_50_ = 15.62 µM and 16.51 µM), respectively, against *Leishmania* promastigotes and *Trypanosoma* epimastigotes [28]. Based on ethnozoological studies (wild chimpanzees were observed to chew young stems of *Vernonia amygdalina)*, antiplasmodial sesquiterpenes vernodalin and vernolide, hydroxyverniladin have been isolated [60]. Oketch-Rabah *et al.* [101] observed that macrocyclic germancrane dilactone 16,17-dihydrobrachycalyxolide from *Vernonia brachycalyx* has both antileishmanial and antiplasmodial activity.

**Table 2:** Therapeutic uses of important plants of the Asteraceae family reported as an antiparasitic

Plant^1^	Plant part used	Pharmacological	Preparation	Organism tested	Context of use	Reference
*Acanthospermum hispidum* DC.	Whole plant	Antileishmanial	Ethanol extract	*Leishmania amazonensis*	*In vitro*	[27]
				*Leishmania braziliensis*		
	Aerial part	Antitrypanosomal	Dichloromethane/ Methanol/ Aqueous	*Trypanosoma brucei brucei*	*In vitro*	[10]

*Achyrocline flaccida* (Weinm.) DC.	Whole plant	Antileishmanial	Ethanol extract	*Leishmania amazonensis*	*In vitro*	[27]

*Ageratina pentlandiana* (DC.) R. M. King & H. Rob.	Leaf	Antileishmanial	Ethanol extract	*Leishmania amazonensis*	*In vitro*	[27]
				*Leishmania braziliensis*	*In vitro*	[69]

*Ageratum* *conyzoides* (L.) L.	Whole plant	Antiparasitic	Organic (hexane, ethyl acetate, chloroform, methanol) and aqueous extracts	*Trypanosoma brucei Trypanosoma brucei rhodesiense Trypanosoma cruzi Leishmania donovani Plasmodium falciparum*	*In vitro*	[98]
	Whole plant	Chagas disease	Aqueous and ethanolic	*Trypanosoma cruzi*	*In vitro*	[149]
	Whole plant	Antileishmanial	Aqueous and ethanolic	*Leishmania amazonensis*	*In vitro*	[149]
	Leaf	Antiparasitic	Aqueous and ethanol extract	*Heligmosomoides bakeri*	*In vitro*	[157]
	Leaf	Antiparasitic	Ethanol extract	*Rhipicephalus microplus*	*In vitro*	[115]

*Artemisia absinthium* L.	Flower	Antiparasitic	Di-ethyl ether essential oil	*Toxocara cati*	*In vivo*	[163]
				*Trypanosoma cruzi*	*In vitro*	[74]
				*Trichomonas vaginalis*		
				*Trypanosoma cruzi *	*In vitro*	[5]
				*Leishmania infantum*		
	Leaf	Schistosomicidal	Dichloromethane	*Schistosoma mansoni*	*In vitro*	[20]

*Artemisia abyssinica* Sch. Bip. ex A. Rich.	Aerial part	Antitrypanosomal	Dichloromethane: Methanol	*Trypanosoma brucei brucei*	*In vitro*	[94]
			Dichloromethane	*Trypanosoma congolense*	*In vivo*	[25]
	Aerial part	Antitrypanosomal	Dichloromethane: Methanol	*Trypanosoma brucei brucei*	*In vitro*	[94]

*Artemisia afra* Jacq. ex Willd.	Leaf	Antitrypanosomal	Dichloromethane	*Trypanosoma brucei rhodesiense / Trypanosoma cruzi.*	*In vitro*	[82]
		Antitrypanosomal	Dichloromethane: methanol	*Trypanosoma brucei brucei*	*In vitro*	[94]
		Antimalarial	Acetone	*Plasmodium falciparum* NF54	*In vitro*	[85]

*Artemisia annua* L.	Aerial part	Antitrypanosomal	Dichloromethane: Methanol	*Trypanosoma brucei brucei*	*In vitro*	[94]

*Artemisia herba-alba* Asso	–	Antileishmanial	Aqueous	*Leishmania tropica*	*In vitro*	[43]

*Baccharis salicifolia* (Ruiz & Pav.) Pers.	Leaf	Antileishmanial	Ethyl acetate extract	*Leishmania braziliensis*	*In vitro*	[27]

*Baccharis uncinella* DC.	Leaf	Antileishmanial	Ursolic acid	*Leishmania infantum*	*In vivo*	[49]

*Bidens* *pilosa* L.	Leaf	Antimalarial	Organic extracts and fractions	*Plasmodium falciparum*	*In vitro*	[13]
		Antimalarial	Organic extracts	*Plasmodium falciparum*	*In vitro*	[102]
		Antimalarial	Organic extracts	*Plasmodium falciparum*	*In vitro*	[151]
		Antimalarial	Organic extracts	*Plasmodium falciparum,*	*in vitro* & *in vivo* (mice)	[63]
				*Plasmodium berghei* NK-65		
		Anthelmintic	Ethanol extract	*Haemonchus contortus*	*In vitro*	**[36]**
		Antileishmanial	Crude extracts	*Leishmania amazonensis*	*In vitro*	[35,49,151]

*Blumea* *lacera* (Burm.f.) DC.	Leaf	Anthelmintic	Alcoholic and aqueous extracts	*Ascaris lumbricoides*	*In vitro*	[119]
				*Pheretima posthuma*		

*Calendula* *officinalis* L.	Flower	Antileishmanial	Methanol (80%)	*Leishmania major*	*In vitro*	[95]
		Antiparasitic	Oleanolic acid and its glycosides	*Heligmosomoides polygyrus*	*in vitro* & *in vivo* (mice)	[145]

*Centipeda* *minima* (L.) A. Braun & Asch.	Whole plant	Antiparasitic	Crude extracts and fractions	*Giardia intestinalis*	*In vitro*	[164]
				*Entamoeba histolytica*		
				*Plasmodium falciparum*		

*Chersodoma jodopappa* Cabrera	Leaf	Antileishmanial	Ethanol extract	*Leishmania amazonensis*	*In vitro*	[27]
				*Leishmania braziliensis*		
	Stem	Antileishmanial	Ethanol extract	*Leishmania donovani*	*In vitro*	[27]

*Cichorium intybus* L.	Leaf	Anthelmintic	Methanol:water	*Ascaris suum*	*In vitro*	[160]
				*Oesophagostomum dentatum*		

*Cnicothamnus lorentzii* Griseb.	Leaf	Antileishmanial	Ethanol extract	*Leishmania amazonensis*	*In vitro*	[27]
				*Leishmania donovani*		
	Stem	Antileishmanial	Ethanol extract	*Leishmania braziliensis*	*In vitro*	[27]

*Conyza albida* Willd. ex Spreng.	Whole plant	Antitrypanosomal	Dichloromethane: methanol	*Trypanosoma brucei rhodesiense, Trypanosoma cruzi*	*In vitro*	[82]

*Conyza podocephala* DC.	Whole plant	Antitrypanosomal	Dichloromethane: methanol	*Trypanosoma brucei rhodesiense,*	*In vitro*	[82]
				*Trypanosoma cruzi*		

*Conyza scabrida* DC.	Leaf	Antitrypanosomal	Dichloromethane: methanol	*Trypanosoma brucei rhodesiense,*	*In vitro*	[82]
				*Trypanosoma cruzi*		

*Echinacea purpurea* (L.) Moench	Whole part	Antileishmanial	Ethanol extract	*Leishmania* sp.	*In vitro*	[114]

*Eclipta* *alba* (L.) Hassk.	Leaf	Antimalarial	Crude extract	*Plasmodium berghei*	*In vivo*	[6]
		Antileishmanial	Crude extract	*Leishmania donovani*	*In vitro*	[138]

*Eclipta* *prostrata* (L.) L.	Leaf	Anthelmintic activity	Ethanol and aqueous extracts	*Pheretima posthuma*	*In vitro*	[11]
	Leaf	Anthelmintic activity	Organic extracts	*Pheretima posthuma*	*In vitro*	[50]
	Leaf	Antileishmanial	Saponin, dasyscyphin C	*Leishmania major, *	*In vitro*	[56]
				*Leishmania aethiopica,*		
				*Leishmania tropica *		
	Whole plant	Anthelmintic activity	Organic and water extracts	*Haemonchus contortus*	*In vitro*	[139]

*Elephantopus* *scaber* L.	Leaf	Antitrypanosomal	Organic extracts and sesquiterpene lactone	*Trypanosoma brucei rhodesiense*	*In vitro*	[165]

*Helichrysum nudifolium* (L.) Less.	Whole plant	Antitrypanosomal	Dichloromethane: methanol	*Trypanosoma brucei rhodesiense,*	*In vitro*	[82]
				*Trypanosoma cruzi*		

*Inula montana* L.	Aerial part	Antileishmanial	Methanol	*Leishmania infantum*	*In vitro*	[73]

*Jasonia glutinosa* (L.) DC.	Aerial part	Antileishmanial	Acetone	*Leishmania donovani*	*In vitro*	[156]

*Kleinia odora* (Forssk.) DC.	Whole plant	Antiparasitic	Ursane, triterpenes of lupane	*Trypanosoma brucei*	*In vitro*	[89]
				*Trypanosoma cruzi*		
				*Leishmania infantum*		
				*Plasmodium falciparum*		

*Munnozia fournetii* H. Rob. (unresolved name)	Leaf	Antileishmanial	Ethanol extract	*Leishmania amazonensis*	*In vitro*	[27]
				*Leishmania donovani*		
	Stem	Antileishmanial	Ethanol extract	*Leishmania braziliensis*	*In vitro*	[27]

*Neurolaena lobate* (L.) R.Br. ex Cass.	Leaf	Antileishmanial	Ethanol extract	*Leishmania mexicana*	*In vitro*	[9]
				*Leishmania braziliensis*		

*Oedera genistifolia* (L.) Anderb. & K.Bremer	Whole plant	Antitrypanosomal	Dichloromethane: methanol	*Trypanosoma brucei rhodesiense, Trypanosoma cruzi*	*In vitro*	[82]

*Ophryosporus piquerioides* (DC.) Benth. ex Baker	Whole plant	Antileishmanial	Ethanol extract	*Leishmania amazonensis*	*In vitro*	[27]
				*Leishmania braziliensis*		

*Pentzia globosa* Less.	Root	Antitrypanosomal	Dichloromethane: methanol	*Trypanosoma brucei rhodesiense /Trypanosoma cruzi*	*In vitro*	[82]

*Perezia multiflora* (Humb. & Bonpl.) Less.	Leaf	Antileishmanial	Ethanol extract	*Leishmania amazonensis*	*In vitro*	[27]
				*Leishmania braziliensis*		
				*Leishmania donovani*		

*Pterocaulon alopecuroideum* Chodat (unresolved name)	Whole plant	Antileishmanial	Ethanol extract	*Leishmania amazonensis*	*In vitro*	[27]
				*Leishmania braziliensis*		
				*Leishmania donovani*		

*Senecio clivicolus* Wedd.	Leaf	Antileishmanial	Ethanol extract	*Leishmania amazonensis*	*In vitro*	[27]
				*Leishmania donovani*		
	Stem	Antileishmanial	Ethanol extract	*Leishmania braziliensis*	*In vitro*	[27]

*Solanecio mannii* (Hook. F) C. Jeffrey	Leaf	Antitrypanosomal	Dichloromethane: methanol	*Trypanosoma brucei brucei*	*In vitro*	[94]

*Sphaeranthus* *indicus* L.	Whole plant	Anthelmintic	Ethanolic and aqueous extracts	*Pheretima posthuma,*	*In vitro*	[134]
				*Ascaridia galli*		
	Leaf	Macrofilaricidal activity	Methanolic	*Setaria digitata*	*In vitro*	[96]

*Stevia yaconensis* Hieron.	Whole plant	Antileishmanial	Ethanol extract	*Leishmania amazonensis*	*In vitro*	[27]
				*Leishmania braziliensis*		
				*Leishmania donovani*		

*Tagetes* *erecta* L.	Root	Antimalarial	Organic and aqueous extracts	*Plasmodium falciparum*	*In vitro*	[41]
	Flower	Anthelmintic	Organic extracts	*Pheretima posthuma*	*In vitro*	[106]

*Tithonia diversifolia* (Hemsl.) A. Gray	Leaf	Antitrypanosomal	Dichloromethane: methanol	*Trypanosoma brucei brucei*	*In vivo*	[103]

*Tridax* *procumbens* (L.) L.	Whole plant	Antileishmanial property	Organic extracts and (3S)-16,17 didehydrofalcarinol	*Leishmania mexicana*	*In vitro*	[75]
			Methanol extract and in combination with *Allium sativum*	*Leishmania mexicana*	*In vivo*	[33]
			Oxylipin, (3S)-16,17-didehydrofalcarinol	*Leishmania mexicana*	*In vitro*	[75]

*Vernonia* *anthelmintica* (L.) Willd.	Whole plant	Anthelmintic	Aqueous and methanolic extracts	*Haemonchus contortus*	*in vitro* & *in vivo*	[45]
	Seed	Anthelmintic	Ethanolic extract	*Haemonchus contortus*	*In vitro*	[44]
	Seed	Anthelmintic	–	*Haemonchus contortus*	*In vivo* (buffaloes)	[93]

*Vernonia* *auriculifera* Hiern	Root	Antitrypanosomal	Dichloromethane	*Trypanosoma brucei rhodesiense*	*In vitro*	[29]

*Vernonia* *hirsute* (DC.) Sch. Bip. ex Walp.	Whole plant	Antitrypanosomal	Dichloromethane: methanol	*Trypanosoma brucei rhodesiense,*	*In vitro*	[82]
				*Trypanosoma cruzi*		

*Vernonia* *mespilifolia* Less.	Leaf	Antitrypanosomal	Dichloromethane: methanol	*Trypanosoma brucei rhodesiense,*	*In vitro*	[82]
				*Trypanosoma cruzi*		

*Vernonia* *natalensis* Oliv. & Hiern	Whole plant	Antitrypanosomal	Dichloromethane: methanol	*Trypanosoma brucei rhodesiense,*	*In vitro*	[82]
				*Trypanosoma cruzi*		

*Vernonia* *oligocephala* Katt	Leaf	Antitrypanosomal	Dichloromethane	*Trypanosoma brucei rhodesiense,*	*In vitro*	[82]
				*Trypanosoma cruzi*		

*Vernonia* *squamulose* Hook. & Arn.	Stem	Antileishmanial	Ethanol extract	*Leishmania amazonensis*	*In vitro*	[27]
				*Leishmania braziliensis*		
				*Leishmania donovani*		

*Werneria nubigena* Kunth	Leaf	Antileishmanial	Ethanol extract	*Leishmania amazonensis*	*In vitro*	[27]
				*Leishmania donovani*		
	Stem	Antileishmanial	Ethanol extract	*Leishmania braziliensis*	*In vitro*	[27]

*Xanthium catharticum* Kunth	Leaf	Antileishmanial	Ethanol extract	*Leishmania amazonensis*	*In vitro*	[27]
				*Leishmania donovani*		
	Stem	Antileishmanial	Ethanol extract	*Leishmania braziliensis*	*In vitro*	[27]

*Xanthium* *strumarium* L.	Leaf	Antitrypanosomal	50% ethanolic extract	*Trypanosoma evansi*	*In vitro* and *in vivo*	[147]
	Fruit	Antimalarial	Methanol: water extract	*Plasmodium falciparum* strain FCR-3	*In vitro*	[153]

1 All taxonomic names were verified in the Global Composite Checklist database (http://compositae.landcareresearch.co.nz/Default.aspx)

Phenols are widely distributed in Asteraceae, and some have the ability to inhibit parasites. Gallic acid and its derivatives inhibit the proliferation of *Trypanosoma cruzi* trypomastigotes *in vitro* [58]. Higher activities were observed for the gallic acid esters ethyl-gallate and n-propyl-gallate, which had EC_50_ values of 2.28 and 1.47 µg/mL, respectively, possibly due to increased lipophilicity. Oketch-Rabah *et al.* [101] reported the antiprotozoal activity from *Vernonia brachycalyx* (2́-epicycloisobrachycoumarinone epoxide and its stereoisomer). Both stereoisomers show similar *in vitro* activities against chloroquine-sensitive (CQ-S) and chloroquine-resistant (CQ-R) strains for *Plasmodium falciparum,* as well as *Leishmania major* promastigotes, with EC_50_ values of 0.11 µg/mL and 0.15 µg/mL for *Plasmodium falciparum,* and 37.1 µg/mL and 39.2 µg/mL for *Leishmania major*, respectively. Like phenols, flavonoids are extensively present in Asteraceae plants. Elford *et al.* [21] demonstrated that methoxylated flavonones artemetin and casticin act synergistically with artemisinin *in vitro* against *Plasmodium falciparum.* Later, exiguaflavanone A and B, isolated from *Artemisia indica* (Asteraceae), were shown to exhibit *in vitro* activity against *Plasmodium falciparum.*

The flavonoids can be classified into several subtypes: flavone (1), flavonol (2), flavanone (3), dihydroflavonol (4), flavan-3-ol (5), flavan-3,4-diol (6), chalcone (a structure with one opened ring), aurone, and anthocyanidine (with a positive charge on oxygen O-1). Except for these basic structures, flavonoids also exist in biflavonoid and glycosidic form in the Asteraceae family. Perez-Victoria *et al.* [122] suggested that flavonoids could affect transport mechanisms in *Leishmania*. The C-terminal nucleotide-binding domain of a P-glycoprotein-like transporter, encoded by the ltrmdr1 gene in *Leishmania tropica* and involved in parasite multidrug resistance (MDR), was overexpressed in *Escherichia coli* as a hexahistidine-tagged protein and purified. The *Leishmania tropica* recombinant domain efficiently bound different classes of flavonoids with the following relative affinity: flavone>flavanone>isoflavone>glucorhamnosyl-flavone. The affinity was dependent on the presence of hydroxyl groups at positions C-5 and C-3, and was further increased by a hydrophobic 1,1-dimethylallyl substituent at position C-8.

Brandio *et al.* [13] first reported the antimalarial activity of crude extracts and their fractions from different species of *Bidens,* and provided evidence that this is due to the presence of polyacetylene and flavonoids. Later, Kumari *et al.* [63] and Tobinaga *et al.* [151] isolated the polyacetylene compound (R)-1,2-dihydroxytrideca-3,5,7,9,11-pentayne from leaf extracts of *B. pilosa,* which showed promising antimalarial activity against *Plasmodium falciparum* (Table 3). Moreover, this compound was tested in an *in vivo* model (mice infected with *Plasmodium berghei* NK-65 strain), and results showed that the compound can decrease the average parasitaemia in red blood cells, but further studies addressing its mechanism are required. The genus *Calendula* is very well studied for its phytochemistry, with triterpene alcohols, triterpene saponins, flavonoids, carotenoids and polysaccharides as the major classes of phytoconstituents. Szakie *et al.* [145] isolated several oleanolic acid glycoside derivatives and tested them against *Heligmosomoides polygyrus;* the wormicidal activity of the oleanolic acid glycosides was superior to that of the aglycone, and the level of activity was dependent on the nature of the sugar side-chain at the C-3 position. The first sugar molecule of the glucuronides, *i.e.*, the glucuronic acid attached to the aglycone, appeared to be vital for the antiparasitic properties of these compounds [145]. *E.* *prostrata* was studied by several scientists for its antiparasitic properties such as antimalarial [6], antileishmanial [56,138], and anthelmintic activities [11,50]. Khanna *et al.* [56] isolated dasyscyphin C from the leaves and proved its antileishmanial activities against *Leishmania major, Leishmania aethiopica *and*Leishmania tropica* (Table 3). A sesquiterpene lactone (deoxyelephantopin) was isolated by Zahari *et al.* [165] from *E. scaber* and proved active against *Trypanosoma brucei rhodesience.* Similarly, *T. procumbens* showed significant antileishmanial activity against promastigotes of *Leishmania mexicana*. The active principle was found to be an oxylipin, namely (3S)-16, 17- didehydrofalcarinol [76].

**Table 3:** List of compounds from Asteraceae commonly reported for their antiparasitic properties.

Plant^1^	Name of the compounds/group	Organism tested	References
*Acanthospermum hispidum* DC.	Sesquiterpenic lactones	*Plasmodium falciparum*	[34]

*Acmella ciliate* (Kunth) Cass.	Spilanthol	*Trypanosoma brucei rhodesiense* and *Plasmodium falciparum*	[137]

*Ageratum* *conyzoides* (L.) L.	Methoxylated flavonoids	*Trypanosoma brucei rhodesiense, Trypanosoma cruzi, Leishmania donovani* and *Plasmodium falciparum*	[98]

*Ambrosia tenuifolia* Spreng.	Psilostachyin	*Leishmania mexicana*	[143]
	Peruvin		

*Ambrosia tenuifolia* Spreng. and *Ambrosia scabra* Hook. & Arn.	Psilostachyin and psilostachyin C	*Trypanosoma cruzi*	[143]

*Artemisia annua* L.	Sesquiterpenes and sesquiterpene lactones	*Plasmodium falciparum*	[127]

*Aspilia africana* (Pers.) C. D. Adams	Thiarubrine A	*Caenorhabditis elegans*	[128]

*Baccharis retusa DC.*	Sakuranetin	*Leishmania sp.*	[40]

*Baccharis uncinella DC.*	Caffeic acid	*Leishmania amazonensis*	[116]
	Pectolinarigenin	*Leishmania braziliensis*	

*Bidens* *pilosa* L.	Polyacetylene	*Plasmodium falciparum*	[63,151]

*Bidens sulphurea* (Cav.) Sch. Bip.	2,6-Di-tert-butyl-4-methylphenol, germacrene D, β-caryophyllene	*Schistosoma mansoni*	[1]

*Calendula* *officinalis* L.	Glycosides of oleanolic acid	*Heligmosomoides polygyrus*	[145]

*Centipeda* *minima* (L.) A. Braun & Asch.	Sesquiterpene lactone, brevilin A	*Giardia intestinalis*	[164]
		*Entamoeba histolytica*	
		*Plasmodium falciparum*	

*Chromolaena odorata* f. odorata	Quercetin-4’-methyl ether	*Plasmodium falciparum*	[23]

*Cichorium intybus* L.	Sesquiterpene lactone	*Haemonchus contortus*	[26]

*Coreopsis lanceolate* L.	1-Phenylhepta-1,3,5-triyne and 5-phenyl-2-(1’-propynyl)-thiophene	*Bursaphelenchus xylophilus* and *Caenorhabditis elegans*	[55]

*Dicoma tomentosa* Cass.	Sesquiterpene lactones	*Plasmodium falciparum*	[48]
		3D7 and W2	

*Dicoma anomala* subsp. *gerrardii* (Harv. ex F. C. Wilson) S. Ortiz & Rodr. Oubiña	Eudesmanolide-type sesquiterpene lactone	*Plasmodium falciparum* D10	[38]

*Eclipta* *prostrata* (L.) L.	Dasyscyphin C	*Leishmania major,*	[56]
		*Leishmania aethiopica*,	
		*Leishmania tropica*	

*Elephantopus* *scaber* L.	Deoxyelephantopin	*Trypanosoma brucei rhodesience,* strain STIB 900	[165]

*Fructus arctii*	Arctigenin and arctiin	*Dactylogyrus intermedius*	[158]

*Heterotheca inuloides* Cass.	7-Hydroxy-3,4-dihydrocadalene,	*Giardia intestinalis*	[129]
	7-hydroxycalamenene		

*Kleinia odora* (Forssk.) DC.	Ursolic acid and derivatives	*Plasmodium falciparum*	[89]
		*Leishmania infantum*	
		*Trypanosoma cruzi*	
		*Trypanosoma brucei*	

*Pentacalia desiderabilis* Cuatrec.	Jacaranone	*Leishmania braziliensis*	[83]
		*Leishmania amazonensis*	

*Porophyllum ruderale* (Jacq.) Cass.	Thiophene derivatives	*Leishmania amazonensis*	[146]

*Sphaeranthus* *indicus* L.	Indicusalactone, (−)-oxyfrullanolide, 7-Hydroxyfrullanolide, squalene, 3,5-di-O-caffeoylquinic acid methyl ester, 3,4-di-O-caffeoylquinic acid methyl ester	*Plasmodium falciparum*	[132]

*Tagetes erecta L.*	2-Hydroxymethyl-non-3-ynoic acid, 2-[2,2’]-bithiophenyl-5- ethyl ester	*Plasmodium falciparum MRC-pf-2*	[41]
		*Plasmodium falciparum MRC-pf-56*	

*Tagetes patula* L. Synonym of *Tagetes* *erecta* L.	α-terthienyl, gallic and linoleic acids	*Heterodera zeae*	[24]

*Tridax* *procumbens* (L.) L.	(3s)-16,17-Didehydrofalcarinol,	*Leishmania mexicana*	[75]
	(3S)-16,17-didehydrofalcarinol	*Leishmania mexicana*	[75]

*Tanacetum parthenium* (L.) Sch. Bip.	Parthenolide	*Leishmania amazonensis*	[150]

*Tithonia diversifolia* (Hemsl.) A. Gray	Sesquiterpenes and sesquiterpene lactones	*Plasmodium falciparum*	[38]

*Trixis antimenorrhoea* (Schrank) Mart. ex Baker	Trixanolide	*Leishmania amazonensis*	[72]
		*Leishmania braziliensis*	

*Vernonia amygdalina* Delile	Sesquiterpenes and sesquiterpene lactones	*Plasmodium falciparum*	[100]

*Vernonia brachycalyx* O. Hoffm.	Sesquiterpene dilactone	*Plasmodium falciparum* (K39, 3D7, V1/S and Dd2)	[101]

*Vernonia angulifolia* DC.	Sesquiterpenes and sesquiterpene lactones	*Plasmodium falciparum*	[121]

*Xanthium macrocarpum* DC.	Xanthanolides (xanthinosin xanthatin, xanthinin,	*Leishmania mexicana*	[65]
	4-epiisoxanthanol,	*Leishmania infantum*	
	4-epixanthanol)		

1 All taxonomic names were verified in the Global Composite Checklist database (http://compositae.landcareresearch.co.nz/Default.aspx)

## Antiparasitic activity of flavonoids and terpenoids documented in Asteraceae

**Figure 1:**
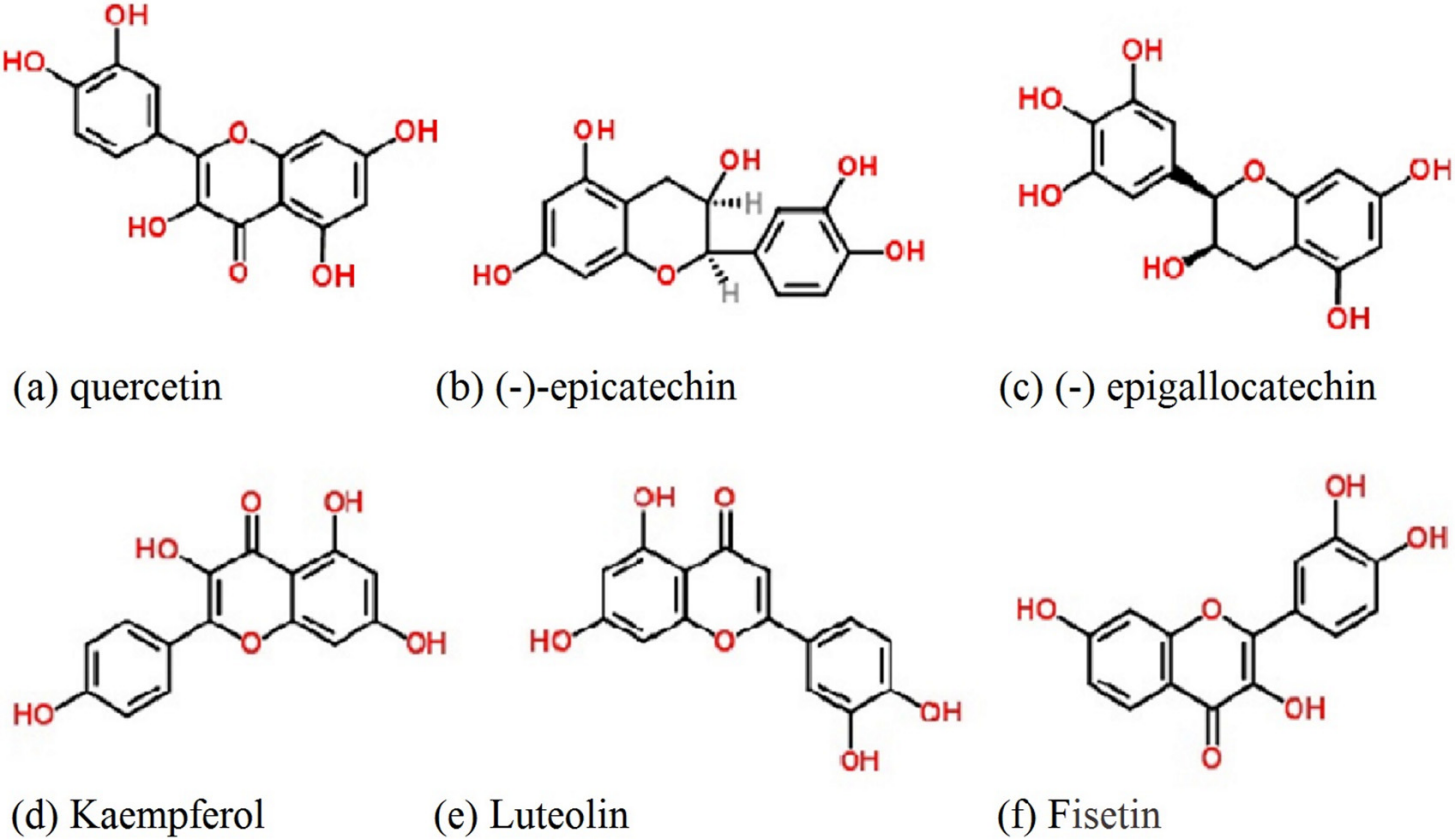
Common flavonoids of the Asteraceae family reported as antiparasitic compounds

Flavonoids are the class of compound of highest occurrence, wide structural diversity, and chemical stability. They have been isolated on a large scale from Asteraceae species and can be used as taxonomic markers at lower hierarchical levels [75]. Flavones and flavonols are common throughout the Asteraceae, *i.e.*, glycosides of apigenin, luteolin, kaempferol, quercetin, flavanone derivatives, (−)-epicatechin and (−)-epigallocatechin (Figure 1). Although there are fewer reports on antigiardial activity in Asteraceae, these compounds from other families are well-studied against *G. lamblia*. From the aerial parts of *Helianthemum glomeratum* (Cistaceae), kaempferol, quercetin, (−)-epicatechin and (−)-epigallocatechin have shown antigiardial activity against *G. lamblia* (*in vitro*), with IC_50_ values of 26.47, 8.73, 1.64 and 8.06 μg/mL, respectively [17]. Structure-activity correlation implies that the 2,3-double bond and 4-keto group of flavones might not be required for antiprotozoal activity since both (−)-epicatechin and (−)-epigallocatechin lack these structural units, yet maintain biological activity (Figure 1). Also, unlike flavones, the benzenediol moiety of (−)-epicatechin and (−) epigallocatechin is not coplanar with the heterocyclic part because C-2 of their flavan-3-ol structure is an sp3 carbon. In addition, there are several reports that glycosylated flavonoids also possess antigiardial activity. Also, a C-3 glycosylated flavone tiliroside [17,79], obtained from *H. glomeratum*, has been shown to possess antigiardial inhibitory activity with an IC_50_ value of 17.36 μg/mL.

Recently, Klongsiriwet *et al.* [57] demonstrated that quercetin and luteolin are highly effective at 250 µM to reduce the *in vitro* exsheathment of *Haemonchus contortus* L3 larvae. Tasdemir *et al.* studied the antitrypanosomal and antileishmanial activities of flavonoids and their analogues *in vitro* and *in vivo*, as well as their (quantitative) structure-activity relationship [148]. They showed that fisetin, 3-hydroxyflavone, luteolin, and quercetin are the most potent antileishmanial compounds against *Leishmania donovani*, with IC_50_ of 0.6, 0.7, 0.8, and 1.0 µg/mL, respectively (Table 4). Moreover, these authors found moderate antitrypanosomal efficacy of these compounds against *Trypanosoma brucei rhodesiense* and *Trypanosoma cruzi*. The authors conclude that 7,8-dihydroxyflavone and quercetin appeared to ameliorate parasitic infections in mouse models, and are potent and effective antiprotozoal agents. Mead and McNair [78] also studied the antiparasitic activity of flavonoids and isoflavones against *Cryptosporidium parvum* and *Encephalitozoon intestinalis*. These authors also found that quercetin and apigenin had activity against *Encephalitozoon intestinalis* at EC_50_ of 15 and 50 mM, respectively, while low activity of luteolin and quercetin was found against *Cryptosporidium parvum*. No inhibition was observed with either rutin or epigallocatechin gallate against either parasite. Lehane and Saliba [66] investigated the effects of a range of common dietary flavonoids on the growth of two strains of the human malaria parasite *Plasmodium falciparum* and concluded that luteolin showed IC_50_ values of 11 ± 1 µM and 12 ± 1 µM for strains 3D7 and 7G8, respectively. Although luteolin was found to prevent the progression of parasite growth beyond the young trophozoite stage, it did not affect parasite susceptibility to the antimalarial drugs chloroquine or artemisinin. Nour *et al.*, [98] found moderate antiparasitic activity of five methoxylated flavonoids viz. 5,6,7,8,5-pentamethoxy-3,4-methylenedioxyflavone (eupalestin), 5,6,7,5-tetramethoxy-3,4-methylenedioxyflavone; 5,6,7,8,3,4,5-heptamethoxy-flavone (5-methoxynobiletine), 5,6,7,3,4,5-hexamethoxy-flavone and 4-hydroxy-5,6,7,3,5-pentamethoxy-flavone (ageconyflavone) against several parasites: *Trypanosoma brucei rhodesiense*, *Trypanosoma cruzi*, *Leishmania donovani* and *Plasmodium falciparum* (Table 4).

**Table 4:** Selected flavonoids and terpenoids (whose presence has been reported in plants of the Asteraceae family) with antiparasitic activity

Flavonoids	Organism tested	Concentration/dose IC_50_	References
Four polyoxygenated flavonoids	*Trypanosoma brucei rhodesiense*	C1: 16 μM, C2: 18 μM, C3: 21 μM and C4: 11 μM	[97]

5,6,7,8,5-Pentamethoxy-3,4-methylenedioxy flavone	*Trypanosoma brucei rhodesiense*;	Tb: 6.67 μg/mL	[98]
	*Trypanosoma cruzi*;	Tc- > 30 μg/mL	
	*Leishmania donovani* and	Ld: > 30 μg/mL	
	*Plasmodium falciparum*	Pf: 4.57 μg/mL	

5,6,7,5-Tetramethoxy-3,4-methylenedioxyflavone	*Trypanosoma brucei rhodesiense*;	Tb: 7.29 μg/mL	[98]
	*Trypanosoma cruzi*;	Tc: 19.5 μg/mL	
	*Leishmania donovani* and	Ld: > 30 μg/mL	
	*Plasmodium falciparum*	Pf: 4.26 μg/mL	

5,6,7,8,3,4,5-Hepta-methoxyflavone	*Trypanosoma brucei rhodesiense*;	Tb: 4.76 μg/mL	[98]
	*Trypanosoma cruzi*;	Tc; 26.4 μg/mL	
	*Leishmania donovani* and	Ld: 5.29 μg/mL	
	*Plasmodium falciparum*	Pf: > 5 μg/mL	

5,6,7,3,4,5-Hexamethoxyflavone	*Trypanosoma brucei rhodesiense*;	Tb: 8.58 μg/mL	[98]
	*Trypanosoma cruzi*;	Tc: > 30 μg/mL	
	*Leishmania donovani* and	Ld: 8.61 μg/mL	
	*Plasmodium falciparum*	Pf: 2.99 μg/mL	

4-Hydroxy-5,6,7,3,5-pentamethoxyflavone (ageconyflavone C)	*Trypanosoma brucei rhodesiense*;	Tb: 3.01 μg/mL	[98]
	*Trypanosoma cruzi*;	Tc: > 30 μg/mL	
	*Leishmania donovani* and	Ld: 3.56 μg/mL	
	*Plasmodium falciparum*	Pf: 3.59 μg/mL	

3, 5, 7, 3’-Tetrahydroxy-4’-methoxyflavone	*Plasmodium falciparum*	–	[23]

Bractein	*Leishmania donovani*	–	[54]

Kaempferol	*Giardia lamblia*	26.47 μg/mL	[17]

Quercetin	*Giardia lamblia*	8.73 μg/mL	[17]

(−)-Epicatechin	*Giardia lamblia*	1.64 μg/mL	[17]

(−)-Epigallocatechin	*Giardia lamblia*	8.06 μg/mL	[17]

Quercetin	*Haemonchus contortus*	250 μg/mL as highest concentration	[57]

Luteolin	*Haemonchus contortus*	250 μg/mL as highest concentration	[57]
	*Leishmania donovani*	0.8 μg/mL	[148]

Quercetin	*Leishmania donovani*	1 μg/mL	[148]

Fisetin	*Leishmania donovani*	0.6 μg/mL	[148]

3-Hydroxyflavone	*Leishmania donovani*	0.7 μg/mL	[148]

Luteolin	*Plasmodium falciparum* 3D7 and 7G8	3D7: 11 μg/mL	[66]
		7G8: 12 μg/mL	

Terpenoids			

Vernodalin	*Plasmodium falciparum*	4 μg/mL	[100]

Vernodalol	*Plasmodium falciparum*	4.2 μg/mL	[100]

Vernolide	*Plasmodium falciparum*	8.4 μg/mL	[100]

Hydroxyvernolide	*Plasmodium falciparum*	11.4 μg/mL	[100]

16,17- Dihydrobrachycalyxolide	*Plasmodium falciparum* (K39, 3D7, V1/S and Dd2)	K39: 4.2 μg/mL	[101]
		3D7: 13.7 μg/mL	
		V1/S: 3 μg/mL	
		Dd2: 16 μg/mL	

Tagitinin C	*Plasmodium falciparum*	0.75 μg/mL	[38]

15-Acetoxy-8 β-[(2-methylbutyryloxy)]-14-oxo-4, 5-cis-acanthospermolide)	*Plasmodium falciparum* 3D7	2.9 μg/mL	[34]

9 α-Acetoxy-15-hydroxy- 8 β-(2-methylbutyryloxy)-14-oxo- 4,5-Trans-acanthospermolide	*Plasmodium falciparum* 3D7	2.23 μg/mL	[34]

3 β-Hydroxyolean-12-en-28-oic acid (oleanolic acid)	*Leishmania amazonensis*	La: > 100 μg/mL	[116,162], [161]
	*Leishmania braziliensis*	–	

3 β-Hydroxyurs-12-en-28-oic acid (ursolic acid)	*Leishmania infantum*	Li: 7.4 μM	[89]
	*Trypanosoma brucei*	Tb: 2.2 μM	
	*Trypanosoma cruzi*	Tc: 8.8 μM	
	*Plasmodium falciparum*	Pf: 29.7 μM	

Indicusalactone	*Plasmodium falciparum*	2.8 μg/mL	[132]

(−)-Oxyfrullanolide	*Plasmodium falciparum*	3.8 μg/mL	[132]

7-Hydroxyfrullanolide,	*Plasmodium falciparum*	2.5 μg/mL	[132]

Squalene	*Plasmodium falciparum*	2.3 μg/mL	[132]

3,5-Di-O-caffeoylquinic acid methyl ester	*Plasmodium falciparum*	2.4 μg/mL	[132]

(3s)-16,17-Didehydrofalcarinol	*Leishmania mexicana*	0.48 μM	[76]

Ursolic acid	*Leishmania amazonensis*	6.4 μg/mL	[162]
	*Leishmania infantum*	*In vivo* 1.0 mg/kg body weight (mice)	[49]

Urs-12-ene-3 β,16 β-diol	*Plasmodium falciparum*	Pf: 9.7 μM	[89]
	*Leishmania infantum*	Li: 9.3 μM	
	*Trypanosoma cruzi*	Tc: 9.9 μM	
	*Trypanosoma brucei*	Tb: 2.3 μM	

3 β,11α-Dihydroxyurs-12-ene	*Plasmodium falciparum*	Pf: 23.9 μM	[89]
	*Leishmania infantum*	Li: 3.2 μM	
	*Trypanosoma cruzi*	Tc: 8.1 μM	
	*Trypanosoma brucei*	Tb: 7.8 μM	

Betulinic acid	*Caenorhabditis elegans*	100 μg/mL	[22]
	*Plasmodium falciparum* W2	2.33 μg/mL	[91]

β-Sitosterol	*Trypanosoma brucei brucei* S427	12.5 μg/mL	[99]

**Figure 2:**
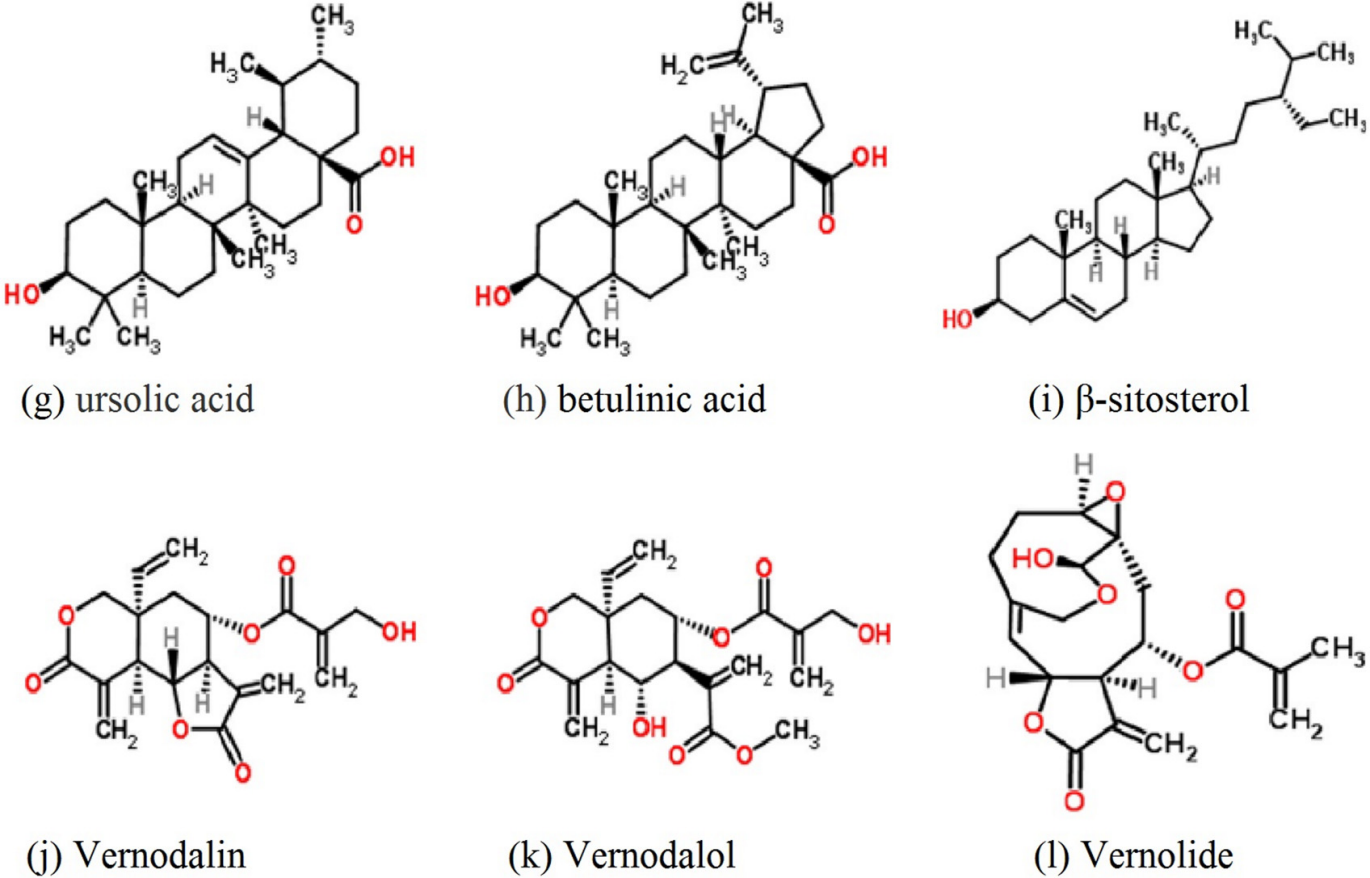
Common terpenoids of the Asteraceae family reported as antiparasitic compounds

Terpenoids are the largest group of phytochemicals as they comprise more than 20,000 recognised molecules. Depending on the number of carbons, terpenoids are divided into classes, starting with sesquiterpenes and continuing with diterpenes, sterols, triterpenes and finally tetraterpenes. Several sesquiterpenes, sterols and triterpenes have been isolated from members of the Asteraceae family. The sesquiterpenes commonly found in leaf extracts from Asteraceae are divided into mono- and bicyclic. The most abundant sterols from Asteraceae are stigmasterol and sitosterol. Sequiterpenes isolated from *Vernonia* spp. have antiparasitic activity against *Plasmodium falciparum*. Four compounds such as vernodalin, vernodalol, vernolide, and hydroxyvernolide (Figure 2), all derived from the leaves of *Vernonia amygdalina,* have potent activity with IC_50_ values of 4, 4.2, 8.4 and 11.4 µg/mL, respectively [60]. Another compound: sesquiterpene dilactone (16,17-dihydrobrachycalyxolide), isolated from the leaves of *V. brachycalyx*, exhibited anti-plasmodial activity against different multidrug-resistant strains of *Plasmodium falciparum* (K39, 3D7, V1/S and Dd2) with IC_50_ values of 4.2, 13.7, 3.0, and 16 µg/mL, respectively [101]. Goffin *et al.* [38] isolated the sesquiterpene lactone: tagitinin C, from the ether extract of *Tithonia diversifolia* and demonstrated antiplasmodial activity against *Plasmodium falciparum* (IC_50_ of 0.75 µg/mL). Becker *et al.* [8] identified urospermal A-15-O-acetate and dehydrobrachylaenolide as the main active compound responsible for the antiplasmodial activity against *Plasmodium falciparum* 3D7 and W2 strains. Ganfon *et al.* [34] investigated the antiparasitic activities of *Acanthospermum hispidum* by isolating two sesquiterpene lactones (15-acetoxy-8 β-[(2-methylbutyryloxy)]-14-oxo-4,5-cis-acanthospermolide), and 9 α-acetoxy-15-hydroxy-8β-(2-methylbutyry-499 loxy)-14-oxo-4,5-transacanthospermolide), both of which exhibited *in vitro* antiplasmodial activity against a chloroquine-sensitive strain (3D7) with IC_50_ values of 2.9 and 2.23 µM, respectively (Table 4).

Among the triterpenes, squalene and lupeol derivatives are the more common ones [67]. Oleanolic acid (3 β-hydroxyolean-12-en-28-oic acid) is a pentacyclic triterpenoid with widespread occurrence in Asteraceae and was found to have antimalarial and antileishmanial activity [89,162]. Recently, Yamamoto *et al.* [162] studied the activity of ursolic acid on *Leishmania amazonensis* (*in vitro* and *in vivo*). They found that ursolic acid eliminated *Leishmania amazonensis* promastigotes with an EC_50_ of 6.4 µg/mL, comparable with miltefosine, while oleanolic acid presented only a marginal effect on promastigote forms at 100 µg/mL. The possible mechanism by which promastigotes were eliminated by ursolic acid was programmed cell death, independent of caspase 3/7, but it was highly dependent on mitochondrial activity. Also, the ursolic acid was not toxic for peritoneal macrophages from BALB/c mice, and it could eliminate intracellular amastigotes, associated with nitric oxide (NO) production. These authors conclude that ursolic acid can be considered an interesting candidate for future testing as a prototype drug for the treatment of cutaneous leishmaniasis. Enwerem *et al.* [22] examined the anthelmintic activity of betulinic acid on *C. elegans* and confirmed its strong anthelmintic activity at 100 µg/mL, comparable to piperazine. Bringmann *et al.* [14] observed that betulinic acid exhibited moderate to good *in vitro* antimalarial activity against asexual erythrocytic stages of *Plasmodium falciparum.* Later, Steele *et al.* [141] concluded that betulinic acid can inhibit *Plasmodium falciparum* (*in vitro*), while *in vivo* experiments failed to reduce parasitaemia (up to 500 mg/mL in a murine malaria model- mice infected with *P. berghei*) and exhibited some toxicity. However, Ndjakou Lenta *et al.* [91] isolated betulinic acid, studied its *in vitro* activity against the *Plasmodium falciparum* W2 strain, and found it to be very potent with an IC_50_ of 2.33 µg/mL. Nweze *et al.* [99] observed that β-sitosterol has modest anti-trypanosomal activity against *Trypanosoma brucei* S427 (*in vitro* IC_50_ 12.5 µg/mL).

## Discussion

In a review on nature-derived drugs, Zhu *et al.* [166] analysed “the ranking of drug-productive plant families based on the ratio of the approved drugs to reported bioactive natural products (including leads of the approved and clinical trials drugs)” and concluded that there are a few top-ranked plant families that produce high numbers of approved drugs among plant-derived medicines. According to Zhu *et al.* [166], Asteraceae is the fourth-largest drug-productive family that has yielded many approved drugs, including antiparasitic, anticancer, antiglaucoma, ant-inflammatory, antihepatotoxic, antiviral and choleretic agents. From 7229 Asteraceae species, 25 clinical drugs (17 approved and 8 in clinical trials) were documented among 1016 searchable drugs [91,99]. There are many FDA-approved nature-derived drugs that originate from Asteraceae as antiparasitics: arteether, artemether, artemisinin, artesunate, coarsucam, co-artemether, dihydroartemisinin and santonin (all from *Artemisia* species). Also, there are a few drugs still in clinical trials as antiparasitics, such as artemisone, arterolane and artelinic acid [92].

Traditional knowledge has proven a useful tool in the search for new plant-based medicines [18]. It has been estimated that the number of traditionally used plant species worldwide is between 10,000 and 53,000 [77]. In India alone, there are about 25,000 plant-based formulations used in folk and traditional medicine [126]. However, only a small proportion have been screened for biological activity [42,140]. Also, there are many specific regions that are less studied than others (only 1% of tropical floras have been investigated) [42]. Odisha’s unique location in Peninsular India has blessed it with an interesting assemblage of floral and faunal diversity (http://odishasbb.nic.in/index.php?lang=en). The state is on the eastern seaboard of India, located between 17° 49’ and 22° 36’ N latitudes and between 81° 36’ and 8°7 18’ E longitudes. It covers an area of 1,55,707 sq km and is broadly divided into four geographical regions, *i.e.* the Northern Plateau (Chhotanagpur), Central River Basins, Eastern Hills and Coastal Plains. The confluence of two major biogeographic provinces of India: the Eastern Ghats (South-West) and Chhotanagpur Plateau (North), make Odisha a rich biodiversity repository with two internationally well-recognised areas: the Similipal Biosphere Reserve and the Chilika Lagoon. The state has a biodiversity board (it is a statutory body established under the Biological Diversity Act of 2002), with a network of 19 wildlife sanctuaries, one national park, one proposed national park, one biosphere reserve, two tiger reserves and three elephant reserves (http://odishasbb.nic.in/index.php?lang=en). Throughout the state, one finds varied and widespread forests harbouring different types of vegetation such as semi-evergreen forests, tropical moist deciduous forests, tropical dry-deciduous forests and littoral and tidal swamp forests, as well as mangroves with unique, endemic, rare and endangered floral and faunal species. The climate of Odisha is characterised by tropical monsoon weather as its coast borders the Bay of Bengal. The weather is classified as summer, monsoon and winter. Searing hot summers with considerably high monsoon downpours and cool, pleasant winters mark the Odisha climate. The average rainfall varies from 1200 mm to 1700 mm across the state, and is the main source of water. Moreover, the state is vulnerable to multiple disasters such as tropical cyclones, storm surges and tsunamis due to its sub-tropical littoral location (http://nidm.gov.in/default.asp). About 62 ethnic tribal communities have been reported in Odisha, of which 13 are known as "Particularly Vulnerable Tribal Groups" (https://en.wikipedia.org/wiki/List_of_Scheduled_Tribes_in_Odisha). Districts such as Kandhamala, Koraput, Malkanigiri, Mayurbhanj, Nabrangpur, Rayagada and Sundargarh have scheduled tribes (officially designated groups of historically disadvantaged people in India) above 50% of the total population. The social, cultural and religious life of aboriginal people is influenced by nature and natural resources available in and around their habitat, which provides their food, medicine, shelter, and various other materials and cultural needs [109,110].

Sasil-Lagoudakis *et al.* [133] published a review entitled “phylogenies reveal the predictive power of traditional medicine in bioprospecting”. Their study, which includes the Asteraceae family, provides unique large-scale evidence that plant bioactivity underlies traditional medicine. According to these authors, “related plants are traditionally used as medicines in different regions, and these plant groups coincide with groups that are used to produce pharmaceutical drugs”. The authors conclude that “phylogenetic cross-cultural comparisons can focus screening efforts on a subset of traditionally used plants that are richer in bioactive compounds, and could revitalise the use of traditional knowledge in bioprospecting”.

Gertrude *et al.* [36] studied the anthelmintic activity of *Bidens* *pilosa* leaf against *Haemonchus contortus* eggs and larvae and concluded that ethanolic extracts have the potential to inhibit the growth of *Haemonchus contortus.* However, further study on the isolation of the active compounds as well as *in vivo* studies are needed. Similarly, antileishmanial activity of *Bidens* *pilosa* leaf was reported by several researchers [31,85], but no compound responsible for this activity has been identified so far. The anthelmintic and wormicidal properties of *Blumea lacera* leaf were evaluated against *Ascaris lumbricoides* and *Pheretima posthuma* [119], but no bioactive compounds have been acknowledged so far. *Calendula officinalis* has been used traditionally by the tribes of Odisha for worm infections. Nikmehr *et al.* [95] found that crude methanolic extracts have antileishmanial activity, but no bioactive molecules have been isolated so far. *Caesulia* *axillaris*, a wetland plant, is used very frequently for the treatment of malaria by the coastal peoples of Odisha. However, despite its long traditional use, its scientific validation as an antiparasitic agent has not been established so far. Also, the phytochemistry of this plant is not well known, except for a few studies on its essential oils. Similarly, plants such as *Centipeda* *minima*, *Sphaeranthus* *indicus* and *Tagetes* *erecta* are used as anthelmintic plants by the tribes of Odisha for the treatment of worm infections. Yu *et al.* [164] found antiparasitic activity of crude extracts of *Centipeda* *minima* and its fractions against *Giardia intestinalis, Entamoeba histolytica* and *Plasmodium falciparum.* Crude extracts of *Sphaeranthus* *indicus* also showed antiparasitic effects on *Ascaridia galli*, *Entamoeba histolytica* and *Setaria digitate* [96,134]. Organic and aqueous extracts of *Tagetes* *erecta* show antiparasitic [41], and anthelmintic properties [106]. However, notwithstanding phytochemical studies, no anti-parasitic compounds have been identified, nor have any *in vivo* studies been conducted so far on these plants. The plant *Elephantopus* *scaber* showed anthelmintic activity against *Pheretima posthuma* in crude extract. However, further study is required to find out the active anthelmintic compounds. Both *in vitro* and *in vivo* studies were carried out and proved the anthelmintic properties of *Vernonia anthelmintica* against *Haemonchus contortus* [103,106,140]. Further study is needed to determine the active anthelmintic compounds. The tribes of Odisha frequently use two other species of *Vernonia*: *V. albicans* and *V. cinerea*. These plants are also interesting for future study to discover active molecules with antiparasitic properties. The antitrypanosomal activity of a crude 50% ethanol extract of *Xanthium* *strumarium* leaves was studied *in vitro* and *in vivo*. The extract exhibited trypanocidal activity against *Trypanosoma evansi*-infected mice [147]. The authors hypothesised that the presence of xanthinin may be responsible for its trypanocidal activity, but further study is needed to definitively identify the antitrypanosomal compound or compounds.

## Conclusion

A search for new antiparasitic drugs has been under way over the past several decades. However, despite the abundant literature, more work is needed to yield potent, commercially available drugs based on natural products. Fortunately, academic drug discovery for neglected diseases has intensified (*e.g.* the Drugs for Neglected Disease Initiative http://www.dndi.org/), and this includes efforts to use natural products (*e.g.* Research Network Natural Products against Neglected Diseases https://www.facebook.com/ResNetNPND/app/435433039823956). Although many Asteraceae species were already studied for different antiparasitic activities, some of the species important in traditional medicines have still hardly been studied for their bioactivity. Therefore, the present review aims to encourage further exploration of their potential bioactivity and particularly their antiparasitic properties, guided by the knowledge on the use of Asteraceae plants by the tribes of Odisha and corresponding traditional uses elsewhere in the world. The work reported here highlights the traditional uses of Asteraceae plants of Odisha for the treatment of parasites. Plants such as *Bidens* *pilosa,* *Blumea lacera,* *Caesulia* *axillaris*, *Centipeda* *minima* and *Sphaeranthus* *indicus* deserve to be studied further, especially concerning their most relevant bioactive properties and significant bioactive compounds that could be purified with state-of-the-art methods.

## Conflict of interest

The authors declare that they have no conflict of interest.
